# Incorporating Point-of-Care Bacterial Fluorescence into a Wound Clinic Antimicrobial Stewardship Program

**DOI:** 10.3390/diagnostics10121010

**Published:** 2020-11-26

**Authors:** Thomas E. Serena

**Affiliations:** SerenaGroup Research Foundation, Cambridge, MA 02140, USA; serena@serenagroups.com

**Keywords:** antimicrobial stewardship, chronic wounds, clinical decision support, diagnostic pathway, fluorescence imaging, wound clinic

## Abstract

Background: In 2014 the World Health Organization (WHO) warned of an emerging world-wide crisis of antibiotic-resistant microorganisms. In response, government and professional organizations recommended that health care systems adopt antimicrobial stewardship programs (ASPs). In the United States, the Centers for Medicare Services (CMS) mandated antimicrobial stewardship in the hospital inpatient setting. Effective 1 January 2020, the Joint Commission required ambulatory centers that prescribe antibiotics, such as wound centers, to institute an ASP. Chronic wounds often remain open for months, during which time patients may receive multiple courses of antibiotics and numerous antimicrobial topical treatments. The wound clinician plays an integral role in reducing antimicrobial resistance in the outpatient setting: antibiotics prescribed for skin and soft tissue infections are among the most common in an outpatient setting. One of the most challenging aspects of antimicrobial stewardship in treating chronic wounds is the inaccuracy of bacterial and infection diagnosis. Methods: Joint Commission lists five elements of performance (EP): (1) identifying an antimicrobial stewardship leader; (2) establishing an annual antimicrobial stewardship goal; (3) implementing evidence-based practice guidelines related to the antimicrobial stewardship goal; (4) providing clinical staff with educational resources related to the antimicrobial stewardship goal; and (5) collecting, analyzing, and reporting data related to the antimicrobial stewardship goal. This article focuses on choosing and implementing an evidence-based ASP goal for 2020. Discussion: Clinical trials have demonstrated the ability of fluorescence imaging (MLiX) to detect clinically significant levels of bacteria in chronic wounds. Combined with clinical examination of signs and symptoms of infection, the MLiX procedure improves the clinician’s ability to diagnose infection and can guide antimicrobial use. In order to satisfy the elements of performance, the MLiX procedure was incorporated into the annual ASP goal for several wound care centers. Clinicians were educated on the fluorescence imaging device and guidelines were instituted. Collection of antimicrobial utilization data is underway.

## 1. Introduction

The world-wide emergence of antibiotic-resistant bacteria endangers the efficacy of antibiotics and increases the morbidity and mortality associated with infectious diseases [1,2,3]. In 2013, the Centers for Disease Control and Prevention (CDC) declared that mankind had entered a “post-antibiotic era [4].” Shortly thereafter, the World Health Organization (WHO) warned of a dire antibiotic resistance crisis [5]. Although antimicrobial resistance may occur naturally over time, the misuse and overuse of antimicrobials has accelerated the process [5]. Antimicrobial stewardship plays a pivotal role in controlling bacterial resistance to antibiotics. Antimicrobial stewardship programs (ASPs), mandated in acute care settings and skilled nursing facilities, have successfully reduced antimicrobial use without sacrificing clinical outcomes [6]. This year, hospital outpatient departments must institute ASPs [7].

The outpatient wound care center features prominently in the development of antimicrobial resistance for several reasons: antibiotic prescriptions written in the outpatient setting are most commonly for skin and soft tissue infections; the typical wound is open for more than three months, during which time the patient may receive repeated courses of antibiotics and topical antimicrobials; and uninfected wounds with excessive inflammation are often misdiagnosed and treated as infected [8,9]. More importantly, the clinical signs and symptoms of infection are inaccurate and unreliable in chronic wounds [10]. Current diagnostic techniques, such as swabs, are fraught with inaccuracies with regard to chronic wounds, and culture reports take days to return to the clinician [11]. In addition, they do not distinguish bacteria present in biofilms from planktonic forms and may not detect anaerobic bacteria or other potential pathogens that are, using standard hospital laboratory techniques, difficult to grow [12]. Quantitative tissue biopsies of the wound bed are more accurate than swabs but require an invasive procedure, and it takes days for the results to return [12].

The MolecuLight procedure (MLiX) detects bacterial fluorescence in real time at the point-of-care [13,14]. The portable non-contact fluorescence imaging device emits violet light at 405 nm that, under darkened room conditions, causes porphyrin-producing bacteria (e.g., *S. aureus)* to fluoresce red while *Pseudomonas aeruginosa* uniquely fluorescence cyan and tissue fluoresces green [15]. Under the violet light, red fluorescence is observed from most common wound pathogens (gram positive, gram negative, aerobes, and anaerobes), including bacteria found in biofilm, though the red fluorescent signal cannot distinguish between biofilm and planktonic bacteria [16]. Prospective clinical trials have established the accuracy of this fluorescence imaging technology to identify the presence of moderate-to-heavy bacterial load with a positive predictive value of >95% [17,18]. In wounds, moderate bacterial load is defined as greater than 10^4^ DFU. There is a robust body of evidence suggesting that bacterial loads at this level or greater inhibit wound healing [19]. Studies have reported high sensitivity and specificity of MLiX in detecting bacterial fluorescence in a variety of wounds including diabetic foot ulcers, venous leg ulcers, pressure ulcers, burns, and surgical and trauma wounds [13,14,18,19,20,21,22]. Information provided by fluorescence imaging on the presence and location of bacteria at loads >10^4^ CFU/g is used to target cleaning, debridement, and appropriate deployment of antimicrobials [14,18].

Effective 1 January, 2020, the Joint Commission requires ambulatory centers, such as wound centers, that prescribe antibiotics to institute an ASP [7]. The Joint Commission lists five elements of performance to achieve this goal. This manuscript details a plan to institute an ASP in outpatient and free-standing wound care centers in the United States as mandated by Joint Commission. This includes the incorporation of point-of-care fluorescence imaging to detect bacterial burden, which will satisfy the performance elements of the ASP mandate.

## 2. Instituting an Antimicrobial Stewardship Program (ASP)

Founded in 1951, the Joint Commission (JC) is a global quality improvement organization that accredits and certifies hospitals in the United States. An ASP plan is now mandated in all hospital outpatient wound care centers. This manuscript outlines a plan to meet JC requirements and collect data on antimicrobial use in a prospective manner.

Hospital outpatient wound care centers treat patients with acute and chronic wounds. The ASP will apply to all patients with wounds seen in the clinic that are suspected of having increased bacterial burden in their wound. The ASP is a mandated quality improvement project; therefore, a specific informed consent for treatment and data collection is not required. Similarly, institutional review board (IRB) is not necessary.

Joint Commission lists five elements of performance (EP) for an ASP on its website:

(1) Identifying an antimicrobial stewardship leader;
(2) Establishing an annual antimicrobial stewardship goal;
(3) Implementing evidence-based practice guidelines related to the antimicrobial stewardship goal;
(4) Providing clinical staff with educational resources related to the antimicrobial stewardship goal;
(5) Collecting, analyzing, and reporting data related to the antimicrobial stewardship goal.

This manuscript proposes an evidenced-based solution to satisfy the five elements of performance (EP) and meet the requirements for an ASP in the outpatient wound clinic. In 2020, SerenaGroup^®^ advanced wound and hyperbaric centers instituted this ASP in all of its centers.

- Element 1 (EP-1)

The stewardship leader should have thorough knowledge of chronic wound care and antimicrobial use as well as an understanding of data collection techniques. The clearest choice to satisfy EP-1, identifying an antimicrobial stewardship leader, is the medical director of the wound care center. Medical directors use their dedicated nonclinical time to direct the program, direct education staff, and assist in data collection.

- Element 2 (EP-2)

There are several possible choices for EP-2, establishing an annual antimicrobial stewardship goal; however, the biggest challenge in nonhealing wounds is the accurate diagnosis of elevated bacterial levels. Today, the clinician relies on clinical signs and symptoms (CSS) in treating bacterial burden in chronic wounds; however, an increasing body of evidence suggests that CSS are unreliable. As a result, controlling antimicrobial use in the outpatient wound care center is problematic if not impossible. To address this issue, the author established a 2020 antimicrobial goal (EP-2) of improving the detection of bacteria in chronic wounds. Increasing the clinician’s ability to detect clinically significant bacterial load should lead to the more judicious use of topical antimicrobials and oral antibiotics.

- Element 3 (EP-3)

Incorporation of the MLiX procedure into wound assessment satisfies EP-3, implementing evidence-based practice guidelines. In the recently published FLAAG clinical trial, the MLiX procedure was found to improve the sensitivity of bacterial detection in nonhealing wounds four-fold. In addition, the MLiX procedure detected elevated bacterial load in 46% of wounds in which clinical signs and symptoms were negative [18]. Identifying and treating elevated bacterial levels prior to the development of overt signs of infection is expected to reduce the number of antibiotic prescriptions written, guide the use of topical antimicrobials, improve wound hygiene, direct debridement procedures, and potentially improve healing rates. A robust body of evidence supports the integration of the MLiX into the ASP plan [13,14,17,18,19,20,21,22].

- Element 4 (EP-4)

Education is the key to satisfying EP-4. This manuscript begins the educational process by outlining the process of establishing an ASP. On-line webinars review the evidence for the accuracy of clinical signs and symptoms in detecting bacterial load and for the proper application of the MLiX procedure. Training and competencies on the MLiX procedure are available both in-person as part of the Challenges in Wound Care course series and online. A post-course test score of 80% and above is required for certification on MLiX operation and image interpretation. Image interpretation is proctored by clinicians experienced with the device.

- Element 5 (EP-5)

Prior to proceeding to EP-5, baseline data on antimicrobial use will be obtained from the participating clinics in a retrospective fashion. The clinics will report the number of antibiotic prescriptions written, antimicrobial use, debridement frequency, and healing outcomes over the preceding 6 months. This information will serve as a baseline for subsequent data for subsequent analysis.

Collecting, analyzing, and reporting data (EP-5) may be the most challenging of the five elements. Many wound clinics do not have established processes to analyze large amounts of data. The easiest solution is for wound clinics to work collaboratively to collect and analyze their ASP data. In the author’s wound clinics, the ASP data will be collected from the center’s electronic health record and entered into a central data base. This is the same procedure currently used for collecting quality metrics. As required by regulation, patients seen in the outpatient wound clinic will make up the study population. 

A central research group will analyze the data quarterly. The predetermined endpoints for this quality initiative are the number of antibiotic prescriptions written per patient and per clinic; the use of topical antimicrobials in the clinic and prescribed; the combination of oral antibiotics and topical antimicrobials; the reduction in bacterial fluorescence in patients over time, and the number of debridement procedures, treatment failures, and healing outcomes. The results will be reported quarterly and benchmarked between the participating centers.

The primary outcome measure will be the comparison of antimicrobial use before and after the implementation of the ASP. Secondary endpoints include changes in the frequency of debridement before and after executing the ASP and changes to healing rates at the prespecified timeframe of 12 weeks for all patients. The use of fluorescence images to follow the changes in bacterial burden over time is an exploratory endpoint.

The planned statistical analysis will include all predetermined variables, comparing data from the retrospective baseline data with data collected 6 months post implementation. The dataset is likely to include in excess of 50,000 cases, so the data are likely to be normally distributed, but this will be verified before comparative analysis begins. Descriptive statistics will be used to summarize each variable, with continuous variables analyzed using independent t-tests (e.g., number of antimicrobial applications) and Chi-Square for categorical data (e.g., bacterial fluorescence on a +/−ve basis). Two-tailed tests will be used, with statistical significance set at *p* < 0.05. Data will be analyzed using the latest version of SPSS available at the time.

Finally, as the data base builds, the research team will be able to conduct comparative effectiveness analysis between debridement procedures, various topical antiseptics, and antibiotics.

## 3. Discussion

Presently, the prescription of topical antimicrobials and antibiotics in the wound clinic is best described as indiscriminate. This is not the fault of the woundologist. The assessment of clinical signs and symptoms of increased bacterial load are not sensitive enough on their own to identify levels of bacteria that may impede wound healing [14,18]. Standard laboratory swab cultures are inaccurate and do not permit real time prescribing [11]. The Joint Commission requires outpatient departments that prescribe antibiotics to institute antimicrobial stewardship programs (ASPs). The inability to accurately diagnose elevated bacterial burden in chronic wounds makes establishing an ASP problematic. ASPs have been proposed for wound centers, with rapid, point-of-care bacterial infection diagnostics highlighted as one potential solution [23]. We concur that a reliable ASP with reproducible data would benefit greatly from the incorporation of a point-of-care diagnostic for infection.

**Figure 1:**
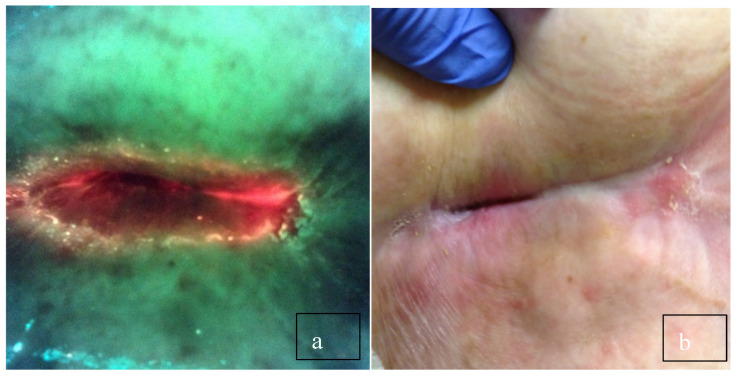
(**a**) Long-standing sacral pressure ulcer negative for bacteria on clinical signs and symptoms. The physician planned to debride the wound and cover it with a plain foam dressing. (**b**) After viewing, the red fluorescence changed the plan to cleansing with hypochlorous acid and applying a topical antimicrobial. Biopsy confirmed >10^5^ CFU/g.

**Figure 2:**
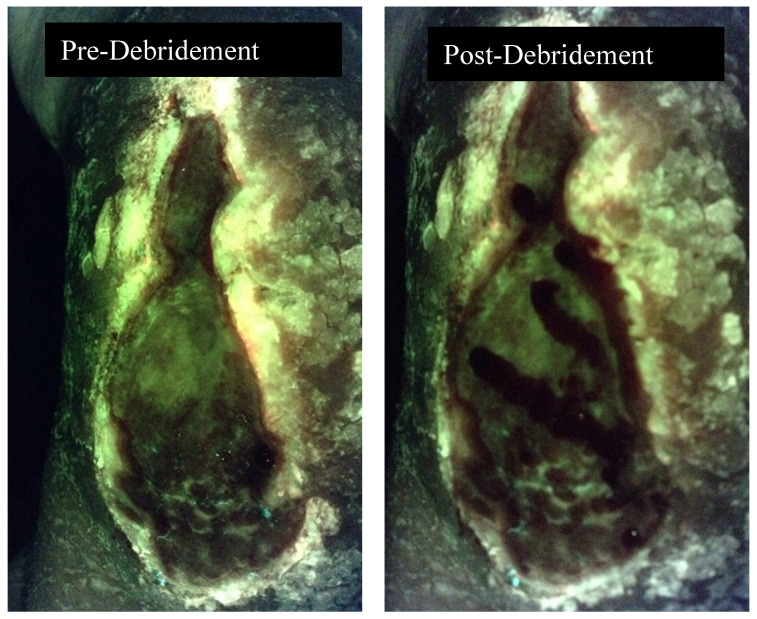
Fluorescence image pre and post debridement of a medical gaiter venous leg ulcer demonstrating the absence of fluorescence following debridement.

**Figure 3:**
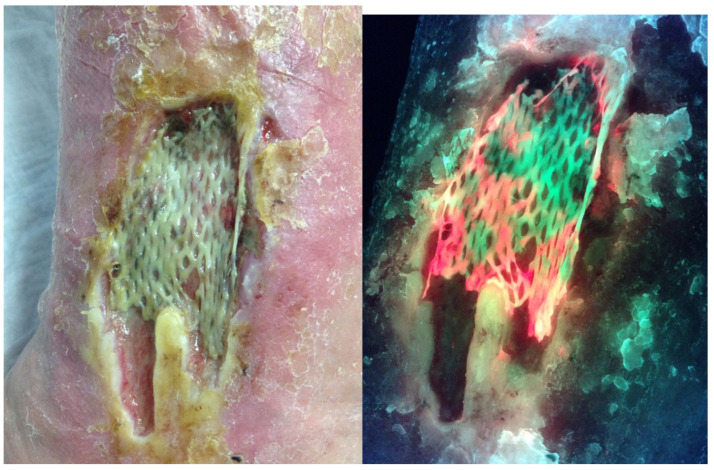
Standard and fluorescence images one week following application of a Cellular- and/or Tissue-Based Product (CTP) on a medical gaiter venous leg ulcer showing red fluorescence associated with the failure.

In a large multicenter clinical trial, investigators changed their antimicrobial treatment 42% of the time based on the fluorescence image (Figure 1) [18]. In some cases, debridement removed bacteria resulting in the absence of fluorescence on the post-debridement image (Figure 2). This was not true for all wounds that were debrided. It has also become clear that the use of the MLiX procedure as part of wound bed preparation could potentially reduce infectious complications associated with the application of cellular or tissue-based products (Figure 3).

This manuscript proposes a straightforward evidence-based method to establish an ASP in the outpatient wound care center, using point-of -care fluorescence imaging to visualize high bacterial load. Data on topical antimicrobial and antibiotic use will be collected from advanced wound care centers across the United States. The pandemic has delayed implementation; however, antimicrobial stewardship will soon become part of standard wound center reporting. The ultimate goal is for wound care centers to share this information. The cumulative data on antimicrobial use will improve patient care and inform future ASP interventions.
